# Correlates of physical activity in adolescence: a study from a developing country

**DOI:** 10.3402/gha.v6i0.20327

**Published:** 2013-05-13

**Authors:** Behjat Shokrvash, Fereshteh Majlessi, Ali Montazeri, Saharnaz Nedjat, Abbas Rahimi, Abolgasem Djazayeri, Davoud Shojaeezadeh

**Affiliations:** 1 Department of Health Education and Promotion, School of Public Health, Tehran University of Medical Sciences, Tehran, Iran; 2 Mental Health Research Group, Health Metrics Research Center, Iranian Institute for Health Sciences Research, ACECR, Tehran, Iran; 3 Department of Epidemiology and Biostatistics, School of Public Health, Tehran University of Medical Sciences, Tehran, Iran; 4 Department of Nutrition and Biochemistry, School of Public Health, Tehran University of Medical Sciences, Tehran, Iran

**Keywords:** adolescent health, physical activity, family support, Asia, Iran

## Abstract

**Background:**

Physical activity is important for adolescent health. The current study aimed to explore factors that predict physical activity among adolescents.

**Methods:**

This was a cross-sectional study of physical activity among a sample of adolescents in Tabriz, Iran. Information on physical activity was collected using a modified version of the Adolescent Physical Activity and Recall Questionnaire (APARQ). In addition, a self-administrated questionnaire was used to collect data on demographic characteristics, perceived family support, and self-efficacy. Both univariate and multivariate logistic regression analyses were performed to examine the association between physical activity and independent variables including gender and psychosocial predictors.

**Results:**

In all, 402 students were studied. The mean age of adolescents was 12.93 (SD=0.49) years; 51.5% were female. The mean time of moderate and vigorous physical activity for all adolescents was 44.64 (SD=23.24) Metabolic Equivalent (MET) min per day. This figure for female adolescents was 38.77 (SD=19.94) MET min per day and for males it was 50.87 (SD=24.88) (P<0.001). The results obtained from multiple logistic regression analysis indicated that female gender (OR=2.59, 95% CI=1.46–4.57, P=0.001) and poor family support (OR=1.10, 95% CI=1.03–1.20, P=0.038) were the most significant contributing factors to low level physical activity in adolescents. Other variables studied did not show any significant results.

**Conclusion:**

The findings from the current study indicated that female adolescents were at risk of lower level of physical activity. In addition, it was found that the lack of family support represented an increased risk for low-level physical activity. It seems that family support should be an integrated part of any health education/promotion programs for improving physical activity among young adolescents in general and for female adolescents in particular.

Sedentary behaviors, lower physical activity, poor nutrition, alcohol consumption, and smoking are increasing both in developed and developing countries (1–5). Regular and adequate physical activity is one of the most important aspects of a healthy lifestyle. It is well known that most behaviors develop during childhood and early adolescence and these behaviors affect health throughout the life course (6, 7) Numerous studies have shown that there are several factors that affect physical activity among adolescents including gender (3, 8, 9), age (2, 3, 10), and support received from family (10–12), friends (13–15), teachers, and coaches (16). For instance, emerging evidence suggest that family support is the most frequent sources of the active lifestyle (17–20). Family support could be either direct or indirect (19–21). A study of direct and indirect effects of social support on adolescents’ physical activity showed that perceived support was associated with physical activity and low self-efficacy (19).

Parents also can strongly influence children's physical activity through role modeling (11, 19, 20), providing practical and emotional support, which may include watching or encouraging them to be active (11, 14, 19) Bauer's findings (19) from a longitudinal study revealed that even parental characteristics influence physical activity in adolescents. Also parental encouragement to be physically active was found to be associated with increased physical activity among males and younger female adolescents. Younger adolescents appeared to be especially influenced by their same-sex parent. These findings suggest that encouragement may be more influential on adolescents’ physical activity habits than parental concerns for fitness. However, in any case adolescents who receive support from either family or friends tend to be more physically active than others (11, 19, 22).

Iran has a young population, yet studies on physical activity among adolescents are scarce. The only national study (5) found that average time of moderate and vigorous physical activity among Iranian adolescents was 28.2 MET min/day, evidently lower than the World Health Organization's recommended time (23). The World Health Organization recommends that children and young people aged 5 to 17 years old should accumulate at least 60 min of moderate to vigorous-intensity physical activity per day. In addition, the existing literature from developing countries suggests that the gender differences in physical activity among adolescents exist and it associates with a number of covariates. Studying physical activity in a Filipino youth sample it was found that 87% of females reported no vigorous activities compared to 18% of males (24). A study from Brazil reported that boys spent more time on sedentary behaviors but also more on physical exercise than girls (25). Similarly a study from Iran showed an identical pattern indicating that boys spent more time both on physical activity and sedentary behaviors than girls (5).

The current study aimed to examine factors contributing to physical activity in Iranian adolescents. Also we were interested in assessing gender differences in physical activity. It was hoped that the findings from this study might add to the existing literature on the topic and perhaps provide necessary information for planning possible future interventions.

## Methods

### Design and data collection

This was a cross-sectional study carried out in Tabriz, Iran, in order to investigate about physical activity in a sample of young students. After obtaining written approval and permission from authorities, schools’ administrators, and one of the parents, a timetable to collect data was developed jointly with the school officials. All students completed the measures individually at classroom on the third week of academic year starting in October 2010. The main investigator (BS) administered the survey questionnaires and was available to answer possible questions. All adolescents had given about 45 min to fill-in the questionnaires.

### Participants

The participants were recruited from four randomly selected government schools (out of 183 schools). There were no restrictions in selecting schools with regard to place and other properties except that we selected two boys’ schools and two girls’ schools. The schools were not matched for any characteristics. Based on the prevalence of physical activity of Iranian adolescents (5), it was estimated that 88 students from each school (boys=176, girls=176, total=352) would provide an enough sample size for comparing gender differences. A study with such a sample size would have a power of 80% at 5% significance level. However, since we surveyed all students in the 7th grade of these four schools, the actual sample size in this study was 402.

### Measures

We used several questionnaires to collect data. All measures underwent preliminary psychometric evaluations. Forty-five male and female students participated in a pilot study. To test reliability the internal consistency of the questionnaires were measured using the Cronbach's α coefficient. Stability was assessed using the intraclass correlation coefficient (ICC) with a two-week interval between two assessments. Face validity was performed to insure that students understand questions and are in-ease in responding to the questionnaires. The Cronbach's α coefficient and the ICC for each measure are indicated as follows.

1. Demographic questionnaire: this was a 10-item questionnaire including questions on age, gender, and items on parental information (age, education, employment, marital status, etc.).
2. Family Affluence Scale (FAS): to identify the socio-economic status of adolescents we used the FAS scale (26). The measure consists of five different items: car ownership (0, 1, 2, 3 or more), computer and laptop ownership (0, 1, 2, 3 or more), number of rooms excluding kitchens and bathrooms (0, 1, 2, 3 or more), number of telephones (0, 1, 2, 3 or more), and having unshared bedrooms (no=0, yes=1). Participants were asked to report the number of items. Then, the FAS score was calculated by summing the responses, giving a score ranging from 0 to 13. Accordingly the FAS score was categorized into three levels: low=0–4, intermediate=5–8, and high=9–13 (Cronbach's α coefficient=0.88, ICC=0.80).
3. Self-efficacy: this was a 10-item questionnaire using questions from two well-known instruments developed by Dishman et al. (27) and Trost et al. (8). Participants were asked: how confident are you that you can increase your physical activity or reduce your sedentary behaviors? Respondents rated the perceived self-efficacy on a 5-point Likert scale (very unsure=1 to very sure=5) giving a possible score ranging from 10 to 50 (Cronbach's α coefficient=0.86, ICC=0.81).
4. Family support specific to physical activity: this was a 17-item questionnaire containing questions about perceived informational family support specific to physical activity (PIFSPA, three items), perceived emotional family support specific to physical activity (PEFSPA, three items), and perceived practical family support specific to physical activity (PPFSPA, 11 items). The questions were derived from instruments developed by Beets et al. and Sallis et al. (13, 28). Participants were asked to indicate: how often does your mother advice, tell/ give you information about benefits of physical activity, and disadvantages of sedentary behaviors (informational support); or how often does your mother encourage you to do physical activity, praise you during physical activity, or watch your participation (emotional support); and how often does your mother do physical activity with you, provide transportation so you can get to a place where you can do physical activity, and provide the necessary sport facilities for you (practical support). There were also three items to assess the negative aspect of practical support: how often does your mother accuse /critic you when doing physical activity, watch TV program/video game playing whenever you like to do. Each respondent rated the perceived support on a 5-point Likert scale (never, rarely, sometimes/usually, always) giving a possible score ranging from 3–15 for informational support, 3–15 for emotional support and 11–55 for practical support, respectively (Cronbach's α coefficient for the total scale=0.82, ICC=0.83).
5. Physical Activity: to assess pattern and different levels of physical activities: we used the modified version of the Adolescent Physical Activity and Recall Questionnaire-APARQ (29). The questionnaire consisted of a number of items on common activities and games that were categorized into light, moderate, and vigorous activities according to the estimated rate of energy expenditure (METs) for each activity as suggested by Anisworth et al. (30). Television viewing, video and computer game playing were considered as sedentary behaviors while moderate intensity activities included all activities and games with 4–6 METs and activities with 7 and greater METs were considered as vigorous-intensity activities. Participants were provided with examples of each class of physical activity and playing. Adolescents were asked for recalling the times, and number of daily physical activities during a given week. Possible responses were coded with 5 min intervals (0 min, 5 min, 10 min, 15 min, 60 min and over). Average daily duration for each class of activity was computed by multiplying frequencies to duration of each activity level divided to 7 (Cronbach's α coefficient for type and time=0.76, ICC=0.87; Cronbach's α coefficient for frequency of common activities performed=0.87, ICC=0.80).

### Analysis

Descriptive statistics including frequency, percentage, mean, and standard deviations were used to explore the data. For comparing the continuous data we used t-test and chi-square was used for comparing the categorical data. Both univariate and multiple logistic regression analyses were performed to examine the association between dependent variable (physical activity) and independent variables including age, gender, mother's age and employment, parental education and marital status, the FAS, sedentary behavior, perceived family support, and self-efficacy. For the purpose of logistic regression the dependent variable (physical activity) was categorized into two levels: equal or greater than 60 min/day (attained the guideline) and less than 60 min/day (did not attain the guideline) (23). The independent variables in multiple logistic regression models were: adolescents’ age, parental characteristics, sedentary behaviors, self-efficacy, and perceived family support. All analyses were performed for whole sample and separately for girls and boys. Data were analyzed using the SPSS statistics software version 11.5.

### Ethics

The ethics committee of Tehran University of Medical Sciences approved the study. The informed written assent was received from all of adolescents. In addition consent was asked for from one of the parents. Adolescents had a chance to withdraw from the study at any time before or during the completion of the questionnaire.

## Results

### The study sample

In all 402 adolescents were entered into the study. The mean age of participants was 12.93 (SD=0.49) years, and 51.5% were female. There were significant differences between boys and girls in some characteristics including their mothers’ education, mothers’ employment, and self-efficacy. Overall only 15.2% of mothers were employed. The characteristics of the study sample are shown in Table 1.

**Table 1:** The characteristics of the study sample

	Total (n=402) No. (%)	Female (n=207) No. (%)	Male (n=195) No. (%)	P
Age (year)				0.452*
≤12	65 (16.1)	35 (16.9)	30 (15.4)	
13	301 (74.9)	157 (75.8)	144 (73.8)	
≥14	36 (9)	15 (7.2)	21 (1.08)	
Mean (SD)	12.93 (0.49)	12.90 (0.47)	12.95 (0.50)	0.302**
Mother's age				0.163*
20–34	120 (29.9)	61 (29.5)	59 (30.3)	
35–39	143 (35.6)	82 (39.6)	61 (31.3)	
40–55	139 (34.6)	64 (30.9)	75 (38.5)	
Mean (SD)	37.4 (5.13)	36.97 (4.50)	37.83 (5.69)	0.091**
Mother's employment				<0.001*
Housewife	341 (84.8)	161 (77.8)	180 (92.3)	
Employed	61 (15.2)	46 (22.2)	15 (7.7)	
Parent's marital status				0.052*
Married	385 (95.8)	202 (97.6)	183 (93.8)	
Widowed	17 (4.2)	5 (2.4)	12 (6.2)	
Mother's education				0.020*
0–12	352 (87.6)	174 (84.1)	178 (91.3)	
>12	50 (12.4)	33 (15.9)	17 (8.7)	
Mean (SD)	10.36 (3.39)	10.99 (3.03)	9.89 (3.62)	<0.001**
Father's education				0.020*
0–12	320 (79.6)	156 (75.4)	164 (84.1)	
>12	82 (20.4)	51 (24.6)	31 (15.9)	
Mean (SD)	10.89 (3.66)	11.48 (3.31)	10.27 (3.90)	<0.001**
Family Affluence Scale				80.158*
Low	61 (15.2)	25 (12.1)	36 (18.5)	
Medium	309 (70.9)	163 (78.7)	146 (74.9)	
High	32 (8)	19 (9.2)	13 (6.7)	
Self-efficacy				
Mean (SD)	32.42 (8.54)	31.05 (8.91)	33.85 (7.88)	0.001**
Score range	10–50	10–50	10–50	
Informational family support				
Mean (SD)	8.48 (2.37)	8.52 (2.43)	8.45 (2.32)	0.767**
Score range	3–15	3–15	3–15	
Emotional family support				
Mean (SD)	8.81 (2.78)	8.71 (2.95)	8.87 (2.60)	0.278**
Score range	3–15	3–15	3–15	
Practical family support				
Mean (SD)	31.21 (6.52)	30.96 (6.62)	31.46 (6.41)	0.447**
Score range	14–54	15–55	14–48	
Sedentary behaviors				0.055*
>120 min/d	263 (65.4)	114 (55.1)	149 (76.4)	
≤120 min/d	139 (34.6)	93 (44.9)	46 (23.6)	

* Derived from chi-square.

** Derived from *t*-test.

### Physical activity among adolescents

Only 22.1% of adolescents reported equal or greater than 60 min of daily physical activity. The mean time of moderate and vigorous physical activity (MVPA) duration on a typical week was about 44.64 (SD=23.24) MET min/day for the whole sample. However, there were significant differences in physical activity between females and males. The mean time of MVPA for females was 38.77 (SD=19.94) MET min/day while this was 50.87 (SD=24.88) MET min/day for males (P<0.001). For instance, hiking and walking to school among females was 8.77 (SD=10.60) MET min/day and it was 9.91 (SD=9.35) for males; or bicycling to school among male students was 15.90 (SD=23.99) MET min/day, whereas this was 5.08 (SD=10.38) for females (P<0.001). There were also significant differences in sedentary and moderate physical activities between females and males. The results are presented in Table 2.

**Table 2:** Daily physical activity among the study sample by gender

	All (n=402)	Female (n=207)	Male (n=195)	P*
Sedentary behaviors (MET minutes/day)				
Mean (SD)	192.83 (89.23)	170.26 (89.20)	218.25 (103.34)	<0.001
Frequency (%) for optimal sedentary behaviors (≤120 minutes/day)	139 (34.6)	93 (44.9)	46 (23.6)	
Moderate physical activity (MET minutes/day)				
Mean (SD)	33.57 (19.98)	31.34 (1.71)	36.03 (1.98)	0.011
Frequency (%) for optimal moderate physical activity (≥60 MET minutes/day)	36 (9.0)	16 (7.7)	20 (10.3)	
Vigorous physical activity MET (minutes/day)				
Mean (SD)	11.03 (9.21)	7.44 (5.83)	14.84 (10.54)	<0.001
Frequency (%) for optimal vigorous physical activity (≥10 MET minutes/day)	180 (44.8)	60 (29.0)	120 (61.5)	
Moderate and vigorous physical activity (MET minutes/day)				
Mean (SD)	44.64 (23.24)	38.77 (19.94)	50.87 (24.88)	<0.001
Frequency (%) for optimal moderate and vigorous physical activity (≥60 MET minutes/day	89 (22.1)	35 (16.90)	54 (27.7)	

* Derived from *t*-test.

### Factors predicting physical activity

The daily physical activity was considered as an outcome measure (dependent variable) and the association between dependent variable and independent variables including gender, age, parental characteristic, self-efficacy, perceived informational, emotional, and practical family support were assessed for whole sample and for girls and boys separately. Overall the results obtained from multiple logistic regression analysis indicated that gender (OR for female gender=2.59, 95% CI=1.46–4.57, P=0.001), and practical family support (OR for low practical family support=1.10, 95% CI=1.03–1.20, P=0.038) were the significant factors contributing to low-level physical activity. Other variables studied did not show any significant results. However, when the analysis was performed for girls and boys separately, the findings were slightly different. For girls emotional family support (OR for low perceived emotional family support=1.02, 95% CI=0.67–0.99, P=0.043), and practical family support (OR for low perceived family support=1.11, 95% CI=1.02–1.24, P= 0.013) were the significant factors contributing to low level physical activity, while for boys informational family support (OR for low perceived informational family support=1.10, 95% CI=0.62–0.90, P= 0.002) was the only significant factor contributing to low level physical activity. The results for whole sample and girls and boys are shown in Table 3.

**Table 3:** Results obtained from logistic regression analysis for low-level physical activity

	Female (n=207)		Male (n=195)		Both sexes (n=402)	
			
	Unadjusted OR (95% CI)	Adjusted OR* (95% CI)	Unadjusted OR (95% CI)	Adjusted OR* (95% CI)	Unadjusted OR (95% CI)	Adjusted OR* (95% CI)
Gender						
Male	NA**	NA	NA	NA	1.0 (ref.)	1.0 (ref.)
Female	NA	NA	NA	NA	2.49 (1.52–4.09)	2.59 (1.46–4.57)
Age (year)						
≤12	1.0 (ref.)	1.0 (ref.)	1.0 (ref.)	1.0 (ref.)	1.0 (ref.)	1.0 (ref.)
13	2.0 (0.61–3.65)	1.01 (0.37–3.28)	2.11 (0.68–1.57)	2.45 (0.97–6.15)	1.69 (0.92–3.12)	1.57 (0.82–3.00)
≥14	4.15 (0.50–14.1)	2.04 (0.17–19.2)	2.50 (0.65–4.21)	2.95 (0.78–11.1)	2.21 (0.78–6.18)	2.27(0.76–6.76)
Mother's age (year)						
40–55	1.0 (ref.)	1.0 (ref.)	1.0 (ref.)	1.0 (ref.)	1.0 (ref.)	1.0 (ref.)
35–39	1.04 (0.25–1.62)	1.10 (0.20–1.88)	1.04 (0.41–1.54)	1.41 (0.58–3.43)	1.10 (0.45–1.41)	1.02 (0.47–1.78)
20–34	1.10 (0.22–1.54)	0.48 (0.15–1.49)	2.02 (0.68–3.42)	1.10 (0.37–1.98)	1.11 (0.49–1.64)	1.20 (0.40–1.40)
Mother's employment						
Housewife	1.0 (ref.)	1.0 (ref.)	1.0 (ref.)	1.0 (ref.)	1.0 (ref.)	1.0 (ref.)
Employed	3.0 (1.21–11.5)	2.36 (0.65–8.52)	2.11 (0.43–5.84)	2.45 (0.34–17.43)	2.35 (0.93–4.45)	2.50 (0.65–5.25)
Parent's marital status						
Married	1.0 (ref.)	1.0 (ref.)	1.0 (ref.)	1.0 (ref.)	1.0 (ref.)	1.0 (ref.)
Widowed	1.10 (0.11–7.51)	1.11 (0.06–8.73)	4.51 (0.57–35.6)	4.47 (0.49–40.3)	2.68 (0.47–28.5)	3.92 (0.36–24.8)
Mother's education (year)						
>12	1.0 (ref.)	1.0 (ref.)	1.0 (ref.)	1.0 (ref.)	1.0 (ref.)	1.0 (ref.)
0–12	2.01 (0.52–5.61)	1.02 (0.21–3.15)	1.11 (0.25–2.53)	2.36 (0.53–10.5)	1.14 (0.54–2.38)	1.30 (0.23–2.10)
Father's education (year)						
>12	1.0 (ref.)	1.0 (ref.)	1.0 (ref.)	1.0 (ref.)	1.0 (ref.)	1.0 (ref.)
0–12	1.30 (0.44–3.89)	1.03 (0.37–2.81)	1.31 (0.56–2.97)	1.26 (0.47–3.38)	1.04 (0.56–1.82)	1.10 (0.55–2.13)
Family Affluence Scale						
High	1.0 (ref.)	1.0 (ref.)	1.0 (ref.)	1.0 (ref.)	1.0 (ref.)	1.0 (ref.)
Medium	2.02 (0.26–9.93)	1.02 (0.13–6.45)	1.11 (0.35–1.84)	1.75 (0.68–4.47)	1.01 (0.54–1.99)	1.03 (0.40–1.71)
Low	1.10 (0.28–2.78)	1.02 (0.12–3.11)	2.03 (0.34–9.88)	1.36 (0.30–6.99)	1.61 (0.52–4.96)	1.41 (0.38–5.40)
Sedentary behaviors						
≤120 min/d	1.0 (ref.)	1.0 (ref.)	1.0 (ref.)	1.0 (ref.)	1.0 (ref.)	1.0 (ref.)
>120 min/d	1.47 (0.69–3.11)	1.12 (0.47–2.65)	1.30 (0.60–2.77)	1.31 (0.55–2.86)	2.02 (0.89–2.56)	1.21 (0.67–2.13)
Self-efficacy	1.01 (0.51–1.99)	1.11 (0.45–2.21)	1.11 (0.96–1.14)	1.02 (0.97–1.60)	1.10 (0.96–1.92)	1.01(0.97–1.93)
IFSPA	1.11 (0.75–1.29)	1.10 (0.80–1.23)	1.10 (0.66–0.88)	1.10 (0.62–.0.90)	1.11 (0.80–0.98)	1.01 (0.79–1.35)
EFSPA	1.10 (0.78–0.99)	1.02 (0.67–0.99)	1.03 (0.82–1.94)	1.07 (0.92–1.25)	1.10 (0.87–1.23)	1.10 (0.86–1.21)
PFSPA	1.01 (0.95–1.19)	1.11 (1.02–1.24)	1.01 (0.96–1.21)	1.10 (0.93–1.19)	1.11 (0.94–1.21)	1.10 (1.03–1.20)

* Adjusted for gender, age, parental characteristics, sedentary behaviors, self-efficacy, and perceived family support (IFSPA=Informational family support specific to physical activity; EFSPA=Emotional family support specific to physical activity; PFSPA=Practical family support specific to physical activity).

** Not applicable.

## Discussion

The findings from this study revealed that gender, and family support were the most significant contributing factors to physical activity among adolescents. Female adolescents were about 2.5 times more likely to have inadequate physical activity per day compared to male adolescents. Our findings are consistent with the previous results where similar conditions were reported by other investigators (3, 5, 13). Gender differences might reflect the fact that girls are more limited in their social activities and receive more restrictions from parents than boys.

The findings from this study indicated that although males were more active than females, at the same time they spent more time for sedentary behaviors including TV viewing, video and computer game playing than females. However, both female and male adolescents spent more times than the recommended levels for sedentary behaviors (<120 min/d) (31). In fact the findings from the current study indicated longer times of sedentary behaviors among our sample compared to the findings from similar studies by Barradas, Wagner, and Davison (9, 32, 33), but shorter times than results reported by other investigators (5, 34, 35). It is argued that access to the electronically devices, amusing games, family conflicts, low knowledge, insufficient parenthood skills to manage adolescents, negative role modeling are possible factors related to the increased sedentary behaviors among adolescents (9, 20, 35).

This study did not find any significant association between age and physical activity and this is dissimilar to findings from previous investigations where age was found to be a significant predictor of physical activity among adolescents (2, 5, 9). We speculate this might be due to limited age range in our study sample.

The Current study did not show that self-efficacy was associated with students’ physical activity. It is argued that self-efficacy might affect adolescent physical activity in two ways directly or indirectly through perceived family support (14, 19, 21), or peer support (14, 15).

Family support showed a significant contributing role to physical activity among adolescents. In fact when the data were analyzed separately, low perceived informational support for males and low practical and emotional support for females were found to be significant predictors of lower level of physical activity. Numerous studies have shown similar result (10, 35–38) confirming that practical and emotional support were the most important type of family support that were associated with adolescents’ physical activity. Even, other studies found that social support had strong effects on adherence to physical activity regardless of having high or low self-efficacy (11, 19, 20).

To increase family support for physical activity several suggestions might be considered. For instance most families are concerned when their young adolescents wish to attend outdoor physical activities. In these occasions families could accompany their children to insure safety and other possible concerns. Another possibility for family support might include provision of light and inexpensive sport facilities at home. Perhaps this could help to prevent young boys and girls of spending too much time watching TV or playing computer games. Families also could ask young adolescents to help in home chores. Particularly families could support young adolescents by walking with them to school, encouraging, even watching them during participation on activities. Finally one might suggest that families with male students should receive information on how and when to promote physical activity in order to be able to support their young boys; while families with female students need to learn to be positive and encouraging about female activities and also provide facilities in order to give more emotional and practical support.

## Limitations

The reliance on self reported physical activity and perceived family support by adolescents is the limitation. In addition we did not collect data on father, sibling, and peers support. Additional research are needed to determine and compare the predictive values of the other potential social support sources including father, sibling, and peers to better understand the influences of parental support on adolescent physical activity and other kind of health behaviors. A small sample and only limited to four government schools is also a limitation of this study. However, since the selection process was random, one might argue that these four schools were typical of the other schools in the region, hence the results might be treated as representative to the adolescents who attended school in that area. We also suggest that future studies also include parameters of cultural measures. Finally, as suggested it is recommended that in the future studies of adolescent physical activity, the physical activity compendium given by Ridley et al. in 2008 (39) should be used instead of that given by Ainsworth et al. (30).

## Conclusion

The findings from the current study indicated that female students were at higher risk of low-level physical activity. However, both female and male students did not meet the recommended physical activity times for adolescents (60 MVPA min/day). The findings also indicated that poor family support represented an increased risk for lack of physical activity. It seems that family support should be an integrated part of any health education/promotion programs for improving physical activity among young adolescents in general and for female adolescents in particular.
